# Peptide Bβ_15-42_ Preserves Endothelial Barrier Function in Shock

**DOI:** 10.1371/journal.pone.0005391

**Published:** 2009-04-29

**Authors:** Marion Gröger, Waltraud Pasteiner, George Ignatyev, Ulrich Matt, Sylvia Knapp, Alena Atrasheuskaya, Eugenij Bukin, Peter Friedl, Daniela Zinkl, Renate Hofer-Warbinek, Kai Zacharowski, Peter Petzelbauer, Sonja Reingruber

**Affiliations:** 1 Department of Dermatology, Medical University Vienna, Vienna, Austria; 2 Fibrex Medical Research & Development GmbH., Vienna, Austria; 3 State Research Center of Virology and Biotechnology “Vector”, Koltsovo, Russia; 4 Center for Molecular Medicine of the Austrian Academy of Sciences, Vienna, Austria; 5 Department of Medicine 1, Division of Infectious Diseases and Tropical Medicine, Medical University Vienna, Vienna, Austria; 6 Department of Vascular Biology and Thrombosis Research, Medical University Vienna, Vienna, Austria; 7 Molecular Cardioprotection & Inflammation Group, Department of Anesthesia, University Hospitals Bristol NHS Foundation Trust, Bristol, United Kingdom; Instituto Oswaldo Cruz and FIOCRUZ, Brazil

## Abstract

Loss of vascular barrier function causes leak of fluid and proteins into tissues, extensive leak leads to shock and death. Barriers are largely formed by endothelial cell-cell contacts built up by VE-cadherin and are under the control of RhoGTPases. Here we show that a natural plasmin digest product of fibrin, peptide Bß15-42 (also called FX06), significantly reduces vascular leak and mortality in animal models for Dengue shock syndrome. The ability of Bß15-42 to preserve endothelial barriers is confirmed in rats i.v.-injected with LPS. In endothelial cells, Bß15-42 prevents thrombin-induced stress fiber formation, myosin light chain phosphorylation and RhoA activation. The molecular key for the protective effect of Bß15-42 is the src kinase Fyn, which associates with VE-cadherin-containing junctions. Following exposure to Bß15-42 Fyn dissociates from VE-cadherin and associates with p190RhoGAP, a known antagonists of RhoA activation. The role of Fyn in transducing effects of Bß15-42 is confirmed in Fyn^−/−^ mice, where the peptide is unable to reduce LPS-induced lung edema, whereas in wild type littermates the peptide significantly reduces leak. Our results demonstrate a novel function for Bß15-42. Formerly mainly considered as a degradation product occurring after fibrin inactivation, it has now to be considered as a signaling molecule. It stabilizes endothelial barriers and thus could be an attractive adjuvant in the treatment of shock.

## Introduction

Capillary leak may be transient, as seen in response to histamine or prolonged as seen in response to thrombin [1]. Extensive leakage often occurs in intensive care patients and is thought to be caused by the exposure of endothelial cells to activated coagulation factors (e.g., thrombin) plus pro-inflammatory stimuli (VEGF, LPS, and others). This results in endothelial cell activation, downregulation of thrombin inhibitors and activation of the small GTPase RhoA. [2]–[6]. By regulating levels of myosin light chain phosphorylation and actin stress fiber formation RhoA controls cell contraction [1], [7]–[9]. Moreover, it results in a rapid activation and redistribution of the integrin-associated focal adhesion kinase (FAK) to the tips of stress fibers [10] thereby giving anchorage support for cell contraction [11]. Cell contraction and rupture of cell-cell contacts results in gap formation and leak [7], [12]–[14]. VE-cadherin is directly connected to the actin-based cytoskeleton and is one of the key molecules integrating signals for opening and tightening of cell junctions [15]–[17].

Peptide Bβ_15-42_ interacts with VE-cadherin [4], [18], [19]. Bβ_15-42_ is a 28 amino acid cleavage product of fibrin. Following thrombin-induced fibrin formation it is released from fibrin E1 fragments by the action of plasmin and represents a sensitive indicator of fibrinolytic activity [20]. This peptide, also called FX06, was shown to prevent myocardial reperfusion injury and to reduce infarct sizes in animal models for myocardial ischemia/reperfusion [4], [20], [21]. Also in a multi-centre phase IIa clinical trial FX06 significantly reduced the size of the necrotic core of infarcts in patients with acute myocardial infarction undergoing primary percutaneous coronary intervention [22].

Parts of this beneficial effect may be explained by the anti-inflammatory properties of FX06 [4]. With VE-cadherin being the molecular target of FX06 we wished to test the hypothesis that this peptide is also interfering with endothelial barrier function. We therefore selected two different models for capillary leak. First, we used an animal model for Dengue shock syndrome (DSS) [23]. DSS -as a hallmark of the disease- presents with slowly progressive vascular leakage developing within days ultimately leading to death [24], [25]. Second, we used a LPS-induced shock model, which rapidly develops leakage within hours [26]. Here we show that FX06 prevents stress-induced RhoA activation. It preserves endothelial barrier function in DSS or LPS-induced shock improves clinical outcomes. As a molecular key for the protective effect of FX06 we identified the src kinase Fyn.

## Results

### FX06 improved survival and reduced capillary leak in Dengue-induced shock

**Figure 1:**
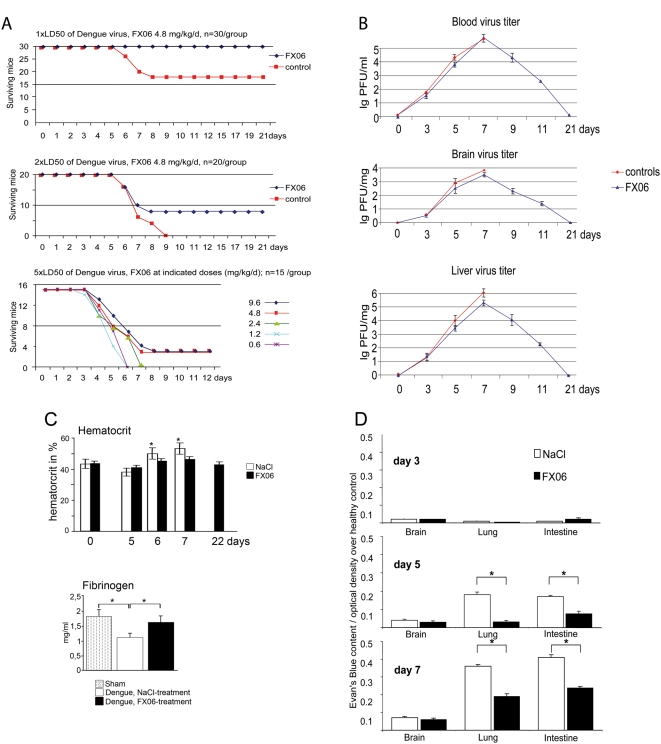
FX06 improved survival following dengue-infection. A. Dengue virus was inoculated *i.p.* at day 0. Starting with day 3, animals were treated with NaCl (100 µl) or indicated doses of FX06 (diluted in 100 µl NaCl). In 1×LD50 and 2×LD50 groups, differences in survival rates between the NaCl and the FX06 groups were significant (p<0.05). B. Virus titers following Dengue infection. Mice were infected with 2×LD50 of Dengue virus i.p and treated with NaCl (controls) or FX06 (2.4 mg/kg bodyweight twice daily). The virus load was quantified by titration of serum or brain lysates onto Vero E6 cell cultures as described Diamond [51] and plaque forming units (PFU) per ml serum or per mg organ were determined. n = 3/data point. All control treated animals were dead at day 9. C. Hematocrit and fibrinogen following Dengue infection. Mice were infected with 2×LD50 of Dengue virus i.p and treated with NaCl (controls) or FX06 (2.4 mg/kg bodyweight twice daily). n = 3/data point, all control mice were dead at day 9, * denotes p<0.05 as compared with controls. D. Organs of 15 animals per time point and group infected with 2×LD50 were analyzed. Treatment started at day 3, mice received NaCl or FX06 (daily dose was 4.8 mg/kg bodyweight in 100 µl NaCl *i.p.*). Samples at day 3 were taken before treatment was initiated. Evan's blue extravasation was measured as described in Methods and data are presented as optical density above that of healthy controls. At days 5 and 7, in lungs and the intestine, the difference in Evan's blue extravasation between the NaCl and the FX06 group was significant (*p<0.05, mean+/−SD).

Dengue shock syndrome (DSS) in humans is characterized by progressive capillary leak [24], [25]. This progressive loss of vascular barrier function is mimicked in our mouse model. Following Dengue infection *i.p.*, mice developed arching backs, ruffling fur, slowed activity, and finally, at days 5–9, they died in a dose-dependent fashion (Fig. 1A). As seen in human patients with DSS [27], our mice presented hemoconcentration and fibrinogen consumption (Fig. 1C) and progressive capillary leak within lungs and the intestine (Fig. 1D). Of not, survival rates correlate with viral titers, which also correlates to the human situation where disease severity correlates with viral loads [28].

Animals treated with FX06 (first treatment on day 3 post infection) had significantly improved survival rates (Fig. 1A), significantly reduced capillary leak within lungs and the intestine (Fig. 1D) and significantly reduced hemoconcentration and fibrinogen consumption (Fig. 1C). Virus loads in serum, liver and brains peaked at day 7 and did not differ between groups (Fig. 1B).

### FX06 reduced capillary leak in systemic LPS shock

**Figure 2:**
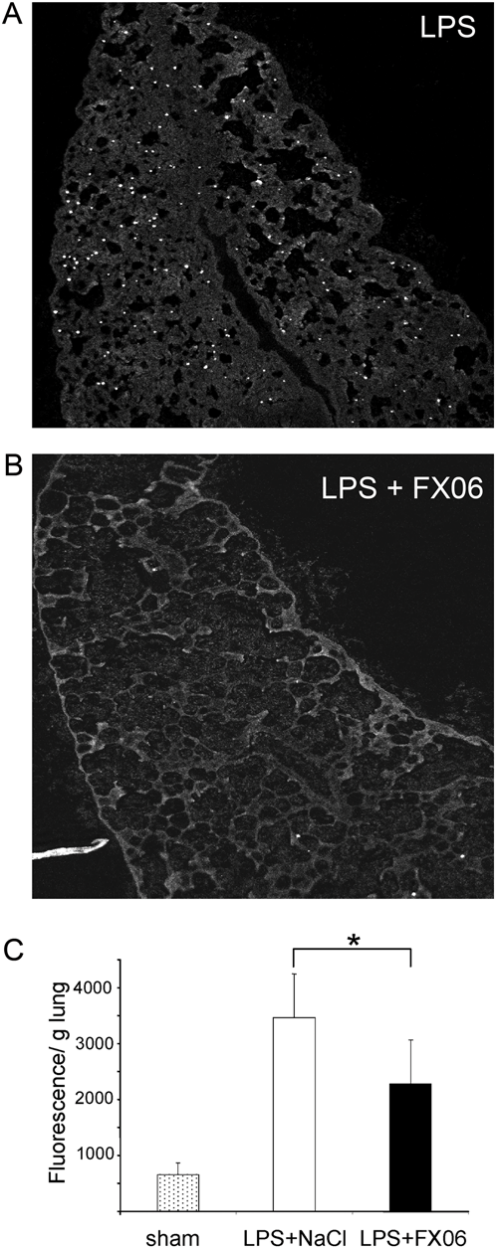
FX06 decreased capillary leak following LPS injection in rats. One hour after LPS injection (*i.v.*, 12 mg/kg), FX06 (2.4 mg/kg) or an equal volume of NaCl (100 µl) were injected *i.v.*; 350 min after LPS injection, FluoSpheres® were injected *i.v.*; 360 min. after LPS injection, rats were sacrificed. The right lung was used for histology and photographed on a laser scan microscope. Examples of images from LPS (A) or LPS+FX06-treated animals (B). From the entire left lung, FluoSpheres® were recovered and fluorescence measured by ELISA as described in Methods (C). The difference between LPS and LPS plus FX06 was significant (n = 11 per group; * denotes p<0.05, mean+/−SD).

To further substantiate the finding that FX06 reduced capillary leak, we injected rats with the gram negative toxin LPS *i.v.*. Capillary leak was assessed by quantifying extravasated 1 µm-seized FluoSpheres® within lungs of LPS-exposed animals. FluoSpheres® within lungs were qualitatively analyzed by histology (example shown in Fig. 2A+B), which was indicative for a reduction of beads in FX06-treated rats (Fig 2B). For quantitative evaluation, FluoSpheres® were recovered from the entire left lungs in each animal by tissue homogenization. Retained fluorescence/g tissue was significantly reduced in FX06-treated animals (Fig. 2C). Further evaluation of organ damage revealed elevated serum liver transaminases in LPS-injected rats (AST mean 1013+/−1016U/l; ALT mean 649+/−835 U/l), which were significantly reduced by FX06 treatment (451+/−202 U/l and 230+/−215 U/l, respectively; n = 10/group; p<0.05). Plasma fibrinogen was 139+/−26 mg/dl in sham treated animals, 44+/−16 in LPS-treated and 54+/−20 mg/dl in LPS+FX06-treated animals.

### FX06 reduced thrombin-induced stress fiber formation

**Figure 3:**
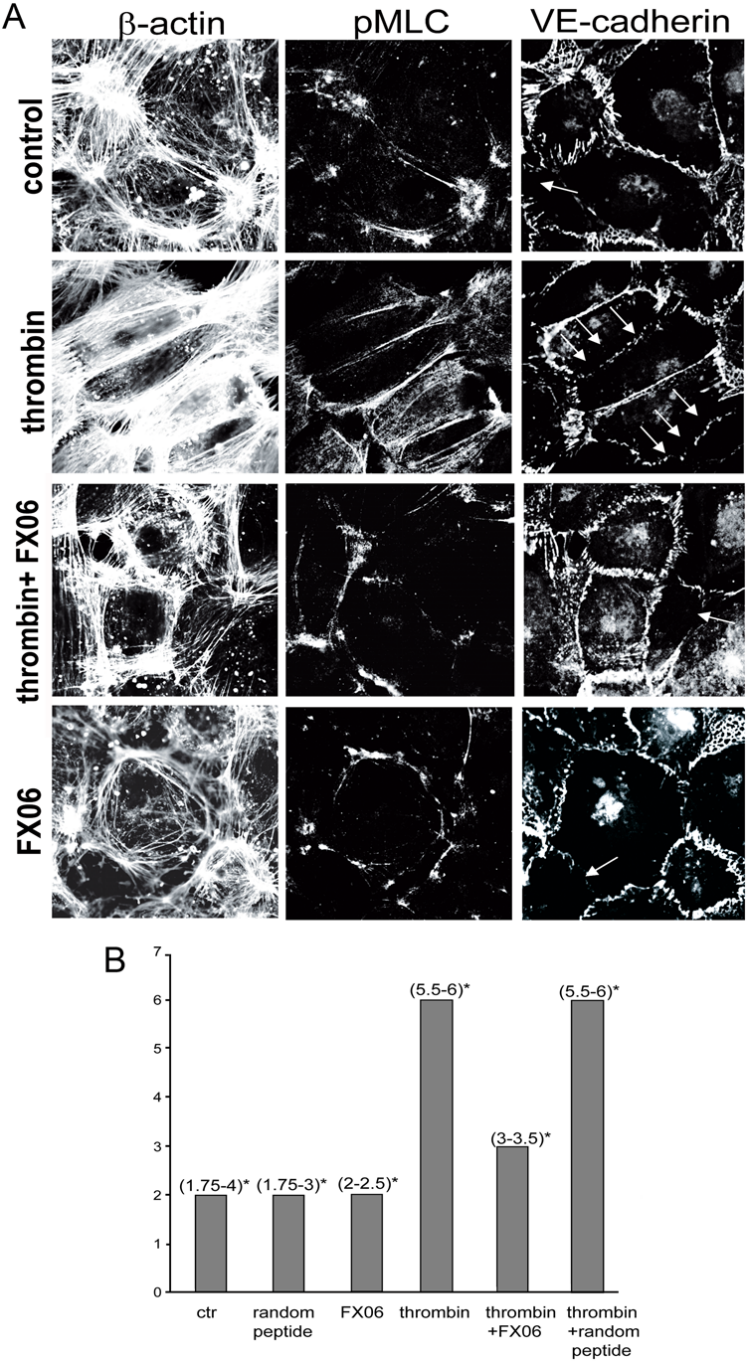
FX06 prevented thrombin-induced actin-bundling and myosin light chain phosphorylation (pMLC). A. Examples of laser scan images of an immunofluorescence triple staining: actin (left) pMLC (middle), and VE-cadherin (right column). Endothelial cells were exposed to thrombin (1 U/ml), FX06 (50 µg/ml) or thrombin+FX06 for 5 min. Following thrombin, actin formed parallel bundles, pMLC co-localized with parallel actin bundles and VE-cadherin formed a discontinuous band. Arrows denote discontinuous VE-cadherin at the cell margins. FX06 (50 µg/ml) prevented these thrombin-induced changes; staining patterns were comparable to those seen in control cells or FX06-treated cells. B. The amount and localization of pMLC, the amount of actin bundling and the continuity of VE-cadherin as exemplified in (A) were quantified in 15 randomly photographed LSM images derived from 3 independent experiments by 2 observers blinded to the condition as described in Methods. Thrombin induced a significant raise in scores as compared to controls (p<0.05) and this was significantly reduced by FX06 (p<0.05, median and interquartile ranges are shown (* denotes the 25% and 75% ranges).

Both dengue and LPS models demonstrated fibrinogen consumption indicative for thrombin activation. Thrombin is known to activate the contractile apparatus of endothelial cells resulting in barrier dysfunction [9], [11], [29]. We therefore first tested effects of the peptide on thrombin-induced stress fiber formation. As shown in Fig. 3A, thrombin induced pronounced parallel bundling of actin fibers (stress fiber formation), increased phosphorylation of myosin light chain and disrupted the continuous lining of VE-cadherin at endothelial surfaces.

In the presence of thrombin plus FX06 only a few stress fibers pervaded the cell. Filamentous actin was mainly located at the cortical band, phosphorylated myosin light chain (pMLC) was found at endothelial cross points and VE-cadherin formed a continuous lining (similar to controls, Fig. 3A). Stress responses were scored (see Methods) and FX06 reduced median thrombin-induced scores by 50% (p<0.05, Figure 3B). The peptide alone had no effect on stress fiber formation and phosphorylation of myosin light chain. Random peptide [4] used as an additional control showed identical results as medium control.

### FX06 inhibited thrombin-induced Focal Adhesion Kinase (FAK) redistribution

**Figure 4:**
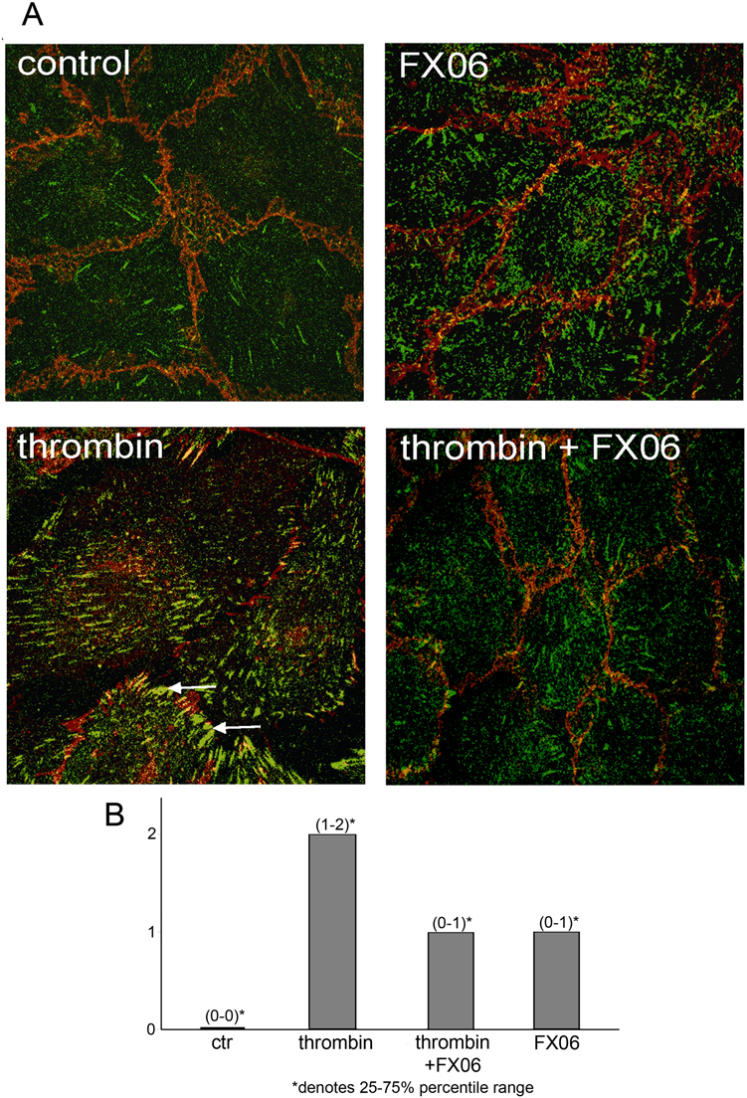
FX06 reduced thrombin-induced FAK activation. A. Immunofluorescence images (anti-ß-catenin Ab in red, anti-phospho-FAK (p397FAK) Ab in green). Endothelial cells were treated as indicated for 5 min. Thrombin induced a robust increase in p397FAK and relocation to cell margins producing a pattern of parallel stripes (arrows) which was not seen in thrombin plus FX06-treated cells. B. The amount and localization of pFAK as exemplified in (A) was scored in 3 independent experiments by 2 observers blinded to the condition as described in Methods. Thrombin induced a significant raise in scores as compared to random peptide-treated cells (*p<0.05) and this was significantly reduced by FX06 (p<0.05), median and interquartile ranges are shown (* denotes the 25% and 75% ranges).

It is well documented that for efficient thrombin-induced cell contraction the activation of focal adhesion kinase (FAK) is required. This provides anchorage to the matrix to allow coordinated cell contraction [11], [30]. As expected [10], we found that thrombin induced a redistribution of pFAK to the tips of stress fibers (Fig. 4A and B). In contrast, in cells treated with thrombin in the presence of FX06, pFAK remained diffusely distributed in the cytosol comparable to controls (Fig. 4A).

### FX06 reduced thrombin-induced RhoA activation

**Figure 5:**
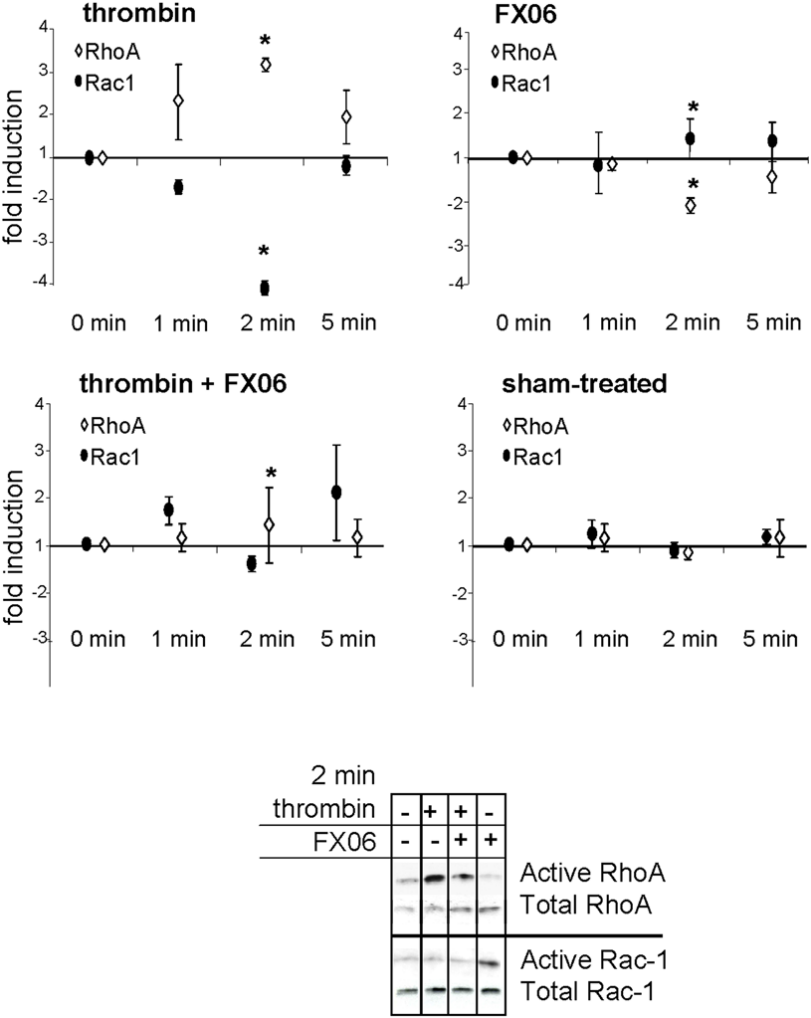
FX06 prevents thrombin-induced RhoA activation. Endothelial cells were exposed to thrombin (1 U/ml), FX06 (50 µg/ml) or thrombin plus FX06 for indicated times. Pull down experiments were performed with GST-coupled Rhotekin (for activated RhoA) or with GST-coupled Pak-1 (for activated Rac1). Graphs depict the mean+/−SD of 4 independent experiments. Thrombin induced significant elevations of RhoA and reductions of Rac1 activity as indicated by asterisks (p<0.05 compared to sham). FX06 alone had the reverse effect (asterisks denote p<0.05 compared to sham). In thrombin plus FX06-treated cells, RhoA activation was completely inhibited (asterisks denote p<0.05 compared to cells treated with thrombin alone). The Western blot is an example of a pull down experiment 2 minutes after stimulation.

Stress fiber formation and myosin light chain activation is under the control of the counteractive balance between RhoA and Rac1 [8], [31]–[34]. As well documented [9], we found thrombin to augment active RhoA and to reduce active Rac1 as determined in pull down assays using GST-coupled Rhotekin and GST-coupled Pak-1 as binding partners for activated GTPases (Fig. 5). Treatment with FX06 alone had reverse effects; it activated Rac1 and decreased the amount of active RhoA (Fig. 5). Combining thrombin with FX06 completely blocked thrombin-induced RhoA activation (Fig. 5).

### FX06 dissociated Fyn from VE-cadherin

**Figure 6:**
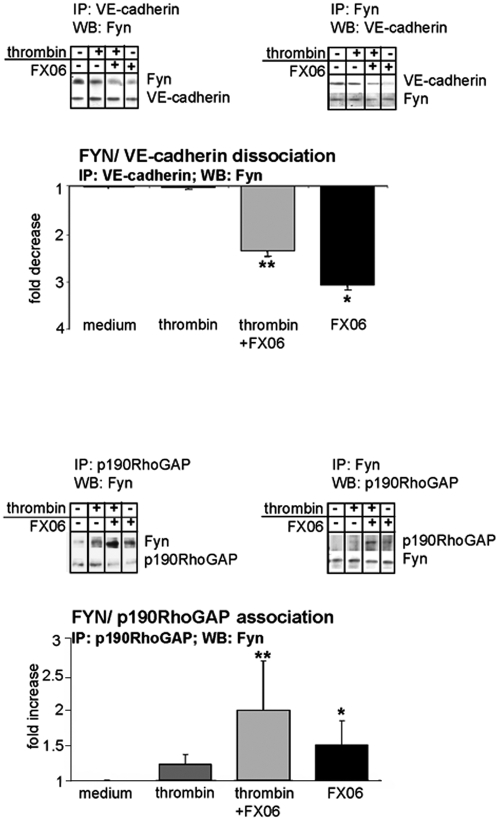
FX06 dissociates Fyn from VE-cadherin and associates Fyn to FAK and p190RhoGAP. Endothelial cells were treated with thrombin, FX06 or thrombin+FX06 for 1 min. Following lysis, proteins were immunoprecipitated (IP) and co-precipitated proteins detected by western blots (WB) as indicated in (A–D). Mean+/−SD of three independent experiments; *p<0.05 compared to medium control, **p<0.05 compared to thrombin; representative Western blots are shown at the right side.

The only yet known endothelial binding partner of the Bß_15-42_ sequence of FX06 is VE-cadherin [4], [18], [19], which has been described to interfere with the counteractive balance of RhoA and Rac1; VE-cadherin/VE-cadherin engagement reduces RhoA and induces Rac1 activity [34], [35]. Since functions of VE-cadherin largely depend on its cytosolic binding partners, we screened effects of the peptide on the composition of the VE-cadherin complex (catenins, VE-PTP, p120cat, c-src, csk and Fyn; data shown for Fyn only). The only significant change observed with FX06 was a rapid dissociation of the src kinase Fyn from VE-cadherin (Fig. 6A). This was paralleled by association of Fyn with FAK with p190RhoGAP (Fig. 6B). Interestingly, thrombin had no effect on the VE-cadherin/Fyn association, whereas the combination of thrombin+FX06 dissociated Fyn from VE-cadherin (Fig. 6A) and associated Fyn with p190RhoGAP (Fig. 6B–D). This raised the possibility that FX06 protected cells from thrombin-induced cell activation in a Fyn-dependent fashion. We tested this in endothelial cells treated with Fyn shRNA which still responded to thrombin, but FX06 was unable to prevent stress fiber formation (Figure S1).

### LPS pneumonitis model with wild-type and Fyn^−/−^ mice

**Figure 7:**
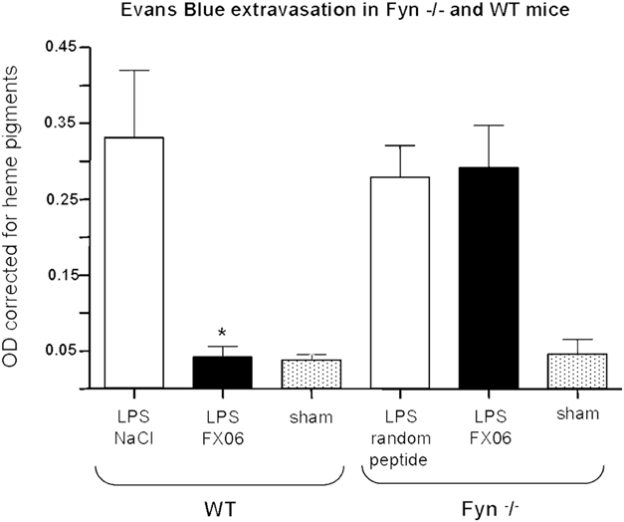
FX06 reduces leak in wild-type but not in Fyn−/− mice. LPS (10 µg) or solvent were instilled intranasally (*i.n.*). FX06 (2×2.4 µg/kg), random peptide or NaCl were injected *i.p.*, the first dose immediately after LPS challenge, the second dose 60 min later. 330 min after LPS challenge, Evan's Blue was injected *i.v.* 30 min later mice were sacrificed and lungs processed as described in the Methods and analyzed for Evan's blue content; mean+/−SD, n = 8 per group. The difference between LPS and LPS+FX06 in wild-type mice was significant, *p<0.05.

As a proof of concept, we tested effects of FX06 in a mouse model of pneumonitis using wild-type and Fyn**^−/−^** mice. Intranasal administration of LPS induced pulmonary inflammation as determined by myeloperoxidase (MPO) activity in lung lysates (4753+/−1018 pg/ml lung). Moreover, LPS induced capillary leak as determined by Evan's blue accumulation within lungs as compared to sham-treated animals. Leak (Fig. 7) and inflammation (3050+/−115 pg/ml lung) were significantly diminished in FX06-treated animals. In contrast, no peptide-related reduction of leak (Fig. 7) or reduction of inflammation (4329+/−1417 and 4157+/−1091 pg/ml respectively) was observed in Fyn^−/−^ mice confirming the role of Fyn in mediating protective effects of FX06.

## Discussion

Data demonstrate that FX06, a plasmin cleavage product of fibrin, reduces capillary leak during sepsis or lung injury. We have shown this in three models of capillary leak. First, the Dengue shock model, where capillary leak started at day 3 and is slowly progressive thereafter and reaches a maximum at day 7. In this model FX06 significantly reduced leak as determined by Evan's blue extravasation and improved survival. Second, we used an intravenous LPS shock model in rats. Importantly, in this non-recovery model barrier function was measured by extravasation of 1 µm-sized beads, which do not extravasate in subacute models like Dengue. Even in this most severe model for a loss of endothelial barrier function FX06 was able to reduce leakage. Third, we used a pneumonitis model induced by LPS inhalation, where FX06 reduced pulmonary leak.

We have shown previously that FX06 forms low affinity interactions with VE-cadherin and has anti-inflammatory properties [4]. We proposed a model where fibrin E1 fragment binding to endothelial VE-cadherin resulted in leukocyte transmigration. The concomitant presence of the fibrin fragment Bbeta15-42 (here called FX06) competed with E1 framents for binding to VE-cadherin thereby reduced leukocyte transmigration. Here we show that FX06 is also a signaling molecule. It antagonizes RhoA activation in vitro and reduces vascular leak in vivo. As reviewed recently by Vestweber [17] mechanisms of leukocyte transmigration and vascular permeability are tightly linked and strongly influenced by VE-cadherin functions. VE-cadherin is a transmembrane molecule crucially involved in the regulation of endothelial barrier function and RhoA activity [16], [35], [36]. FAK reorganisation, actin stress fiber formation and RhoA activation are established signs of stress-induced vascular hyperpermeability [6], [9], [37], [38].

VE-cadherin is organized in a multimeric protein complex called adherens junction. Both, the phosphorylation status of VE-cadherin and the composition of this protein complex are altered in response to factors interfering with vascular barrier function. Among proteins known to be associated with cadherins are catenins, VE-PTP, p120cat, c-src, csk or Fyn. Thus, in a next step we analyzed whether FX06 altered the composition of the VE-cadherin complex. Among proteins co-precipitated with VE-cadherin, only Fyn was significantly affected. The addition of FX06 to endothelial cells caused an immediate dissociation of Fyn from VE-cadherin.

Fyn is a broadly expressed regulatory src kinase. Fyn knock-out mice are viable and have no apparent phenotype [37], [39]–[41], most likely due to compensation by other src kinase family proteins [42]. Also overexpression of Fyn has little effects on the phosphorylation status of other proteins [43], [44]. Not much is known about the function of Fyn in endothelial cells with one exception, the anti-angiogenic effect of thrombospondin requires Fyn [45]. Fyn has been shown to associate with FAK [40], [43], [46] and p190RhoGAP [35], [47]. We here show that Fyn associates with the VE-cadherin-containing adhesion complex and that FX06 dissociates Fyn from VE-cadherin and associates Fyn to p190RhoGAP, known to inhibit RhoA activation thereby preventing cell contraction and maintaining endothelial barrier function [48]. In this context it is of note that FX06 also prevented redistribution of FAK to the tips of stress fibers. It is well documented that for efficient cell contraction FAK is required. FAK provides anchorage to the matrix to allow coordinated cell contraction [11], [30]. Thus, inhibition of FAK relocation might also contribute to the ability of FX06 to maintain barrier function. Anyway, our immunoprecipitation studies postulated that Fyn is a switchpoint for mediating effects of FX06 on endothelial barrier function. To prove this assumption, we used Fyn^−/−^ mice in a pneumonitis model, where LPS induced capillary leak in both, wild-type and knock-out mice, but the protective effect of FX06 was seen in wild-type animals only. We have confirmed the role of Fyn cell culture. FX06 was unable to prevent stress fiber formation in cells infected with Fyn shRNA.

Of note, FX06 also reduced pulmonary inflammation in wild type mice. Given our primary hypothesis that FX06 prevents inflammation by competing with fibrin E1 fragments for the binding to VE-cadherin, we expected that FX06 would diminish inflammation also in Fyn−/− mice also. However, this was not the case. At this point there is no clear answer to this issue, but it can be assumed that inflammatory cells require both, the adhesive step as well as the opening signal to VE-cadherin-based junctions to transmigrate into tissues [17]. In case of FX06, the hierarchy of events (competition with E1 fragments or signaling through Fyn) that prevent inflammation is not established.

There is an interesting analogy between the effects of FX06 on cadherin-based junctions and the physiological effect of homotypic VE-cadherin/VE-cadherin engagement: both results in a reduction of activated RhoA. In the case of cadherin engagement, this is mediated by a yet not identified src kinase family member [35], in case of FX06, this depends on Fyn. In conclusion, we show that FX06 is a signaling molecule that stabilizes endothelial barriers. Combined with the previously described anti-inflammatory effects of FX06 [4], this peptide appears as an attractive adjuvant in the treatment of inflammatory disorders associated with a break down of vascular barrier function.

## Methods

### Ethics statement

All procedures were carried out in accordance with the *Guide for the Care and Use of Laboratory Animals* (NIH Publication No. 86-23). All experiments were approved by local committees and regional governments on animal experimentation: “Vector” Bioethical Committee (IACUC A5505-01), Russia; Medical University Vienna and Vienna government (MA58).

### Peptides and Proteins

FX06 (Bβ_15-42_; GHRPLDKKREEAPSLRPAPPPISGGGYR, 3039.4D) and random peptide (DRGAPAHRPP RGPISGRSTP EKEKLLPG [4]) were produced by solid-phase peptide synthesis and purified with reversed-phase high performance liquid chromatography using nucleosil 100-10C18 columns (Lonza, Brussels and piChem Forschungs- und Entwicklungs-GmbH, Graz). Thrombin (human) was from Sigma-Aldrich.

### Animal models

#### Dengue virus infection

Experiments were performed in the BSL-3 facility (SRC VB ‘Vector’, Russia. The dengue virus DEN-2 (strain P23085) was adapted to adult BALB/c mice (haplotype H-2d) by numbers of sequential intracerebral (*i.c.*) through suckling BALB/c mice followed by intraperitoneal (*i.p.*) passages through immune competent mice at different ages as described [23]; GenBank #AY927231. Six week old male BALB/c mice (14–16 g) infected with this virus *i.p.* develop fever, thrombocytopenia, hemoconcentration and virus-induced death in a dose-dependent fashion [23], [49]. Treatment with FX06 or NaCl was initiated at day 3 following virus infection (*i.p.*). The daily doses were split into two halves, one injected i.p in the morning the other in the evening. Mice were monitored for signs of morbidity and mortality at least twice daily until day 21. Under methoxyflurane anaesthesia blood was taken from the orbital sinus. Capillary leak was determined at days 3, 5 and 7 by *i.v.* injection of 200 µl of 2% Evan's blue in NaCl. One hour later mice were anesthetized and then perfused with 5 ml PBS through the left cardiac ventricle. Organs were removed and Evan's Blue extravasation was quantified as described [50]. Briefly, following tissue homogenization, centrifugation at 5000 g for 30 min, supernatants were analyzed for absorbance at 620 nm. For comparison, Evan's blue was injected into age matched mice neither infected with dengue nor injected with FX06. Leak was calculated as OD values above that of healthy controls.

The virus load was quantified by titration of serum or brain lysates onto Vero E6 cell cultures as described Diamond [51] and plaque forming units (PFU) per ml serum or per mg organ were determined.

#### Lipopolysaccaride shock

Male Spraque Dawley rats (250–280 g; Himberg, Austria) were anesthetized with 100 mg/kg sodium thiopentone (Sandoz). Body temperature was kept constant with a homoeothermic blanket. The trachea and the right jugular vein were cannulated. Following fluid replacement (500 µl 0.9% saline *i.v.*) animals were allowed to stabilize for 15 min. Then, 12 mg/kg LPS (E. coli serotype 0.127:B8; Sigma-Aldrich) was injected *i.v.* One hour later, 2.4 mg/kg FX06 or NaCl were injected *i.v.* as a bolus. 350 min after LPS injection, each rat received FluoSpheres® polystyrene microspheres, 1.0 µm, yellow-green fluorescent *i.v.* (1.2×10^8^/kg bodyweight), labeled with a yellow-green fluorescent dye (Invitrogen Molecular Probes). Six hours after LPS injection, rats were sacrificed and lungs removed. From the left lung, FluoSpheres® were recovered by washing, digestion in KOH and subsequent fluorescent dye extraction according to manufacturer instructions. Fluorescence was determined at Ex 485/Em 538 nm. The right lung was fixed in 4% paraformaldehyde, dehydrated in ascending alcohol concentrations (w/o using xylol) and embedded in paraplast. FluoSpheres® were counted in 5 µm sections in 10 high power fields per sample blinded to the conditions.

#### Lipopolysaccaride pneumonitis

Female 29S7/SvEvBrd*C57BL/6 wild-type or female 29S7/SvEvBrd*C57BL/6 Fyn^−/−^ mice (The Jackson Laboratory) were anesthetized by inhalation of isoflurane (Abbott Laboratories). LPS (10 µg, E. coli O55:B5; Sigma-Aldrich) diluted in 50 µl NaCl was instilled intranasally (*i.n.*), controls received NaCl only. Then, FX06 (2×2.4 mg/kg), random peptide or NaCl were injected *i.p.*, the first dose immediately after LPS challenge, the second 60 min later. 330 min after LPS challenge, mice were anesthetized with ketamine (Pfizer) and Evan's Blue (50 mg/kg; Sigma) was injected *i.v.*; 30 min later, mice were sacrificed, lungs were flushed via the right ventricle using 5 ml cold PBS (pH 7.4), removed and stored in liquid nitrogen. Frozen lungs were homogenized in 300 µl PBS at 4°C followed by incubation in formamide (Calbiochem) at 60°C for 16 h. Absorbance of supernatants was measured at A_620_ and A_720_. Tissue Evan's Blue content was corrected for heme pigments (A_720_) and calculated in comparison to a standard curve according to the formula: A_620_(corrected) = A_620_−(1.426×A_720_+0.030).

### Immunofluorescence-staining of endothelial cells in culture

Human Umbilical Vein Endothelial Cells (HUVEC) isolated from umbilical cords derived from the delivery room of the Medical University of Vienna, used at passages 2 to 6, were grown to confluence on chamber slides (Nunc) in IMDM (GIBCO) containing 20% fetal calf serum, ECGS (50 µg/ml, Promocell) and heparin (5 U/ml) w/o or with FX06 (50 µg/ml), thrombin (1 U/ml) or thrombin+FX06 for indicated times. Following fixation in 4% paraformaldehyde, slides were stained with the following first step antibodies diluted in PBS containing 0.1% TritonX-100 and 1% bovine serum albumin: rabbit anti-phospho-myosin regulatory light chain (3 µg/ml; Chemicon Int.), mouse anti-VE-cadherin (TEA1/31, 1 µg/ml; Immunotech), rabbit-anti-β-catenin (1 µg/ml; Sigma-Aldrich) and mouse anti-phospho focal adhesion kinase (pY397, 0.5 µg/ml; BD Transduction Laboratories). Second step Abs were Cy5-labeled goat anti-mouse IgG (Jackson Laboratories) and Alexa488-labeled anti-rabbit IgG (Invitrogen-Molecular Probes). To visualize actin, sections were incubated with TRITC-labeled phalloidin (0.5 µg/ml; Sigma-Aldrich). Sections were then analyzed by a confocal laser scan microscope (LSM 510, Zeiss) with a pinhole of one airy unit, resulting in a section thickness of 0.8 µm.

Evaluation of stress response was performed by 2 independent observers. Figure 3B shows summation of scores, which were determined as follows.

- parallel actin bundles; absent = 0, discrete bundling = 1, parallel fibers = 2
- myosin light chain phosphorylation; only at junction cross points = 0, minimal colocalization with stress fibers = 1, strong colocalization with stress fibers = 2
- VE-cadherin membrane staining; continuous = 0, single discontinuous sites/cell = 1 discontinuous staining = 2

FAK staining (Fig. 5A) was scored as follows: weak and cytoplasmic = 0, strong and cytoplasmic = 1, strong and at tips of stress fibers = 2.

### RhoA and Rac1 Pull Down

HUVEC grown to confluence were incubated with FX06 (50 µg/ml), thrombin (1 U/ml) or thrombin+FX06 for indicated times or were left untreated. Active RhoA was pulled down by using Rho Assay Reagent (GST-coupled Rhotekin, Upstate) and active Rac1 by using Rac1/Cdc42 Assay Reagent (GST-coupled Pak-1, Upstate) according to manufactures instructions. Bound proteins were separated on a 15% polyacrylamid gel and blotted on Nitrocellulose-Membrane (Bio-Rad). RhoA was detected with anti-RhoA antibody (clone55; Upstate). Rac1 was detected with anti-Rac1 antibody (clone23A8; Upstate). Total RhoA and Rac1 contents were determined in western blots performed from the same lysates before pull downs were performed.

### Immunoprecipitation and Western blotting

HUVEC grown to confluence were stimulated with FX06 (50 µg/ml), thrombin (1 U/ml) or thrombin+FX06 for indicated times or were left untreated. After washing with PBS (4°C), cells were scrapped into Tris-lysis buffer containing 1% TritonX-100, 1% NP-40 and a protease and phosphatase inhibitory cocktail (Sigma-Aldrich). Cells were kept for 20 min on ice and vortexed every 5 min. Following centrifugation, supernatants were harvested and added to 50 µl sepharose beads (Sigma-Aldrich), which were preincubated with the indicated antibodies. Beads were agitated for 2 h at 4°C, washed and incubated with 2× sample buffer at 95°C for 5 min. Beads were removed by centrifugation and supernatants placed onto 10% polyacrylamide gels to separate precipitated proteins. Following blotting onto PVDF (Bio-Rad) membranes, washing with TBS/0.5% TWEEN (TBST), blocking with 1% BSA/TBST for 1 h at RT, membranes were incubated with the indicated antibodies in 1% BSA/TBST over night at 4°C. For detection, HRP-labeled goat anti-mouse or anti-rabbit antibodies (Bio-Rad) in TBST were used and bound Abs were visualized by chemiluminescence (ECL-system, Amersham Corp.) and recorded on film.

For Fyn - p190RhoGAP co-precipitation experiments, membrane fractions were isolated by using the Compartmental Protein Extraction Kit CNMCS (K201301-1-6, Biochain Institute) according to the manufacturer's instructions. Protein concentrations were adjusted to 1 mg protein and samples were added to 5 µg anti-p190RhoGAP antibody (Sigma-Aldrich) or 5 µg anti-Fyn antibody (Santa Cruz) and incubated at 4°C for 12 h. 60 µl Agarose A beads (preincubated with 5% BSA) were added and agitated for 12 h at 4°C. Following washing, beads were incubated with 2× sample buffer at 95°C for 5 min. Beads were removed by centrifugation and supernatants placed onto 10% polyacrylamide gels as described above.

Data analysis was done with Dolphin-1D Gel Analysis Software (Wealtec). Protein bands were identified automatically by the program and analyzed by comparing the optical densities. Coprecipitated protein bands were normalized to proteins precipitated with the respective antibody.

### Statistics

T test with Bonferroni's correction was used. For survival curves (Fig. 1) we used Kaplan Meier estimates. For figures 3 and 4, non parametric statistics were used, median and interquartile ranges are shown and statistics are calculated by Kuskal-Wallis test.

## Supporting Information

Figure S1 Knock down of Fyn with lentiviral siRNA smart vector in cultured HUVEC; Fyn siRNA and control siRNA were purchased from Dharmacon and used according to the manufacturers instructions. For infection 2 MOI/cell were used. Control staining for Fyn (green) and F-actin (red) in scrambled (A) vs. specific siRNA infected cells (B). C–F: Cytoskeleton formation (red) and pFAK (green) distribution in scrambled (C, D) vs. specific siRNA infected cells (E, F). Cells were treated with 1U Thrombin (C–F) without FX06 (C, E) and with 50 µg/ml FX06 (D, F) for 1 min.
(12.04 MB TIF)
