# Correction: Peptide Bβ_15-42_ Preserves Endothelial Barrier Function in Shock

**DOI:** 10.1371/annotation/9ae032a2-c48d-46d9-9f8f-d3f401714e42

**Published:** 2009-06-17

**Authors:** Marion Gröger, Waltraud Pasteiner, George Ignatyev, Ulrich Matt, Sylvia Knapp, Alena Atrasheuskaya, Eugenij Bukin, Peter Friedl, Daniela Zinkl, Renate Hofer-Warbinek, Kai Zacharowski, Peter Petzelbauer, Sonja Reingruber

There was an error in affiliation assignment for author Sylvia Knapp. This author should also be affiliated with #5, Department of Medicine 1, Division of Infectious Diseases and Tropical Medicine, Medical University Vienna, Vienna, Austria. 

