# Supplementary figures and images for: Peptide Bβ15-42 Preserves Endothelial Barrier Function in Shock
Source: PLoS One. 2009 Apr 29;4(4):e5391. doi: 10.1371/journal.pone.0005391 (PMC2670535; doi:10.1371/journal.pone.0005391)

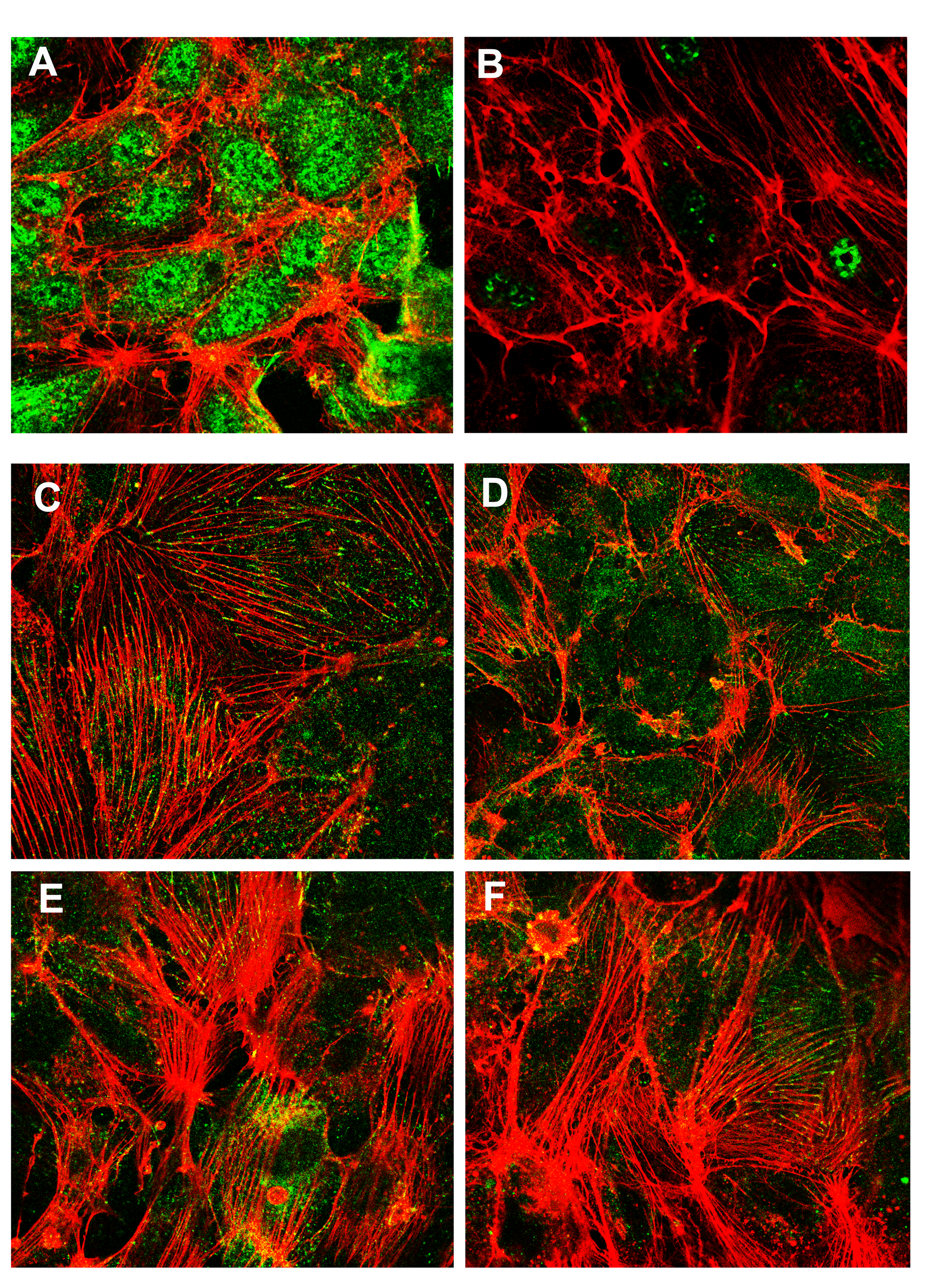

Supplement: Figure S1 — Knock down of Fyn with lentiviral siRNA smart vector in cultured HUVEC; Fyn siRNA and control siRNA were purchased from Dharmacon and used according to the manufacturers instructions. For infection 2 MOI/cell were used. Control staining for Fyn (green) and F-actin (red) in scrambled (A) vs. specific siRNA infected cells (B). C–F: Cytoskeleton formation (red) and pFAK (green) distribution in scrambled (C, D) vs. specific siRNA infected cells (E, F). Cells were treated with 1U Thrombin (C–F) without FX06 (C, E) and with 50 µg/ml FX06 (D, F) for 1 min. (12.04 MB TIF) [file pone.0005391.s001.tif]
